# Systems biology approach to exploring the effect of cyclic stretching on cardiac cell physiology

**DOI:** 10.18632/aging.103465

**Published:** 2020-08-05

**Authors:** Chien-Cheng Chen, Tzyy-Yue Wong, Tzu-Yun Chin, Wen-Hsien Lee, Chan-Yen Kuo, Yi-Chiung Hsu

**Affiliations:** 1 Department of Cardiology, Show Chwan Memorial Hospital, Changhua, Taiwan; 2 International Center for Wound Repair and Regeneration National Cheng Kung University, Tainan, Taiwan; 3 Department of Biomedical Sciences and Engineering, National Central University, Taoyuan, Taiwan; 4 Graduate Institute of Clinical Medicine, College of Medicine, Kaohsiung Medical University, Kaohsiung, Taiwan; 5 Department of Internal Medicine, Kaohsiung Municipal Hsiao-Kang Hospital, Kaohsiung Medical University, Kaohsiung, Taiwan; 6 Department of Internal Medicine, School of Medicine, College of Medicine, Kaohsiung Medical University, Kaohsiung, Taiwan; 7 Department of Research, Taipei Tzu Chi Hospital, Buddhist Tzu Chi Medical Foundation, New Taipei, Taiwan

**Keywords:** next-generation sequencing, cyclic stretching, cardiac cell, functional enrichment

## Abstract

Although mechanical forces are involved in pressure-overloaded cardiomyopathy, their effects on gene transcription profiles are not fully understood. Here, we used next-generation sequencing (NGS) to investigate changes in genomic profiles after cyclic mechanical stretching of human cardiomyocytes. We found that 85, 87, 32, 29, and 28 genes were differentially expressed after 1, 4, 12, 24, and 48 hours of stretching. Furthermore, 10 of the 29 genes that were up-regulated and 11 of the 28 that were down-regulated after 24 h showed the same changes after 48 h. We then examined expression of the genes that encode serpin family E member 1 (SERPINE1), DNA-binding protein inhibitor 1 (ID1), DNA-binding protein inhibitor 3 (ID3), and CCL2, a cytokine that acts as chemotactic factor in monocytes, in an RT-PCR experiment. The same changes were observed for all four genes after all cyclic stretching durations, confirming the NGS results. Taken together, these findings suggest that cyclical stretching can alter cardiac cell physiology by activating cardiac cell metabolism and impacting cholesterol biosynthesis signaling.

## INTRODUCTION

Mechanical stretching affects many cellular functions, including proliferation, differentiation, and survival [1, 2]. Furthermore, cyclic stretching promotes differentiation, survival, and migration in mesenchymal stem cells [3, 4]. Bin Fang et al. showed that cyclic stretch promoted survival, proliferation, adhesion, and migration, while prolonged stretching promoted aging, in adipose-derived stem cells (ADSCs) [5]. Other mechanical stimuli, including shear forces, tissue stiffness, and tissue stretch, affected stem cell fate and differentiation of human-induced pluripotent stem cells (hiPSCs) into cardiomyocytes [6–8]. Furthermore, *in vitro* cyclic stretching enhanced the growth of adult stem cells, human pluripotent cells, and cardiomyocytes by regulating cell contractility and sarcomere maturation [9]. However, increased stretching strain caused cardiac hypertrophy by increasing sarcomeric growth [10]. These data suggest that mechanical stretching plays important roles in physiology and pathophysiology.

A previous study indicated that mechanical stretch induced a cardiac hypertrophic gene program in rat ventricular myocyte cells [11]. Here, we comprehensively characterized the time course of mechanical stretch-activated gene expression in a human cardiomyocyte cell line using a dynamic culture system. We also identified pathways enriched in genes that were differentially expressed after cyclic stretching. Finally, we confirmed NGS expression results for four genes associated with cholesterol biosynthesis and inflammatory response in an RT-PCR experiment.

## RESULTS

### Cyclic stretching alters gene expression profiles

Mechanical stimulation influences cell orientation, which consequently affects cell growth, differentiation, and many cellular functions. The effect of cyclic stretching on gene transcription was determined by stretching AC16 human cardiomyocyte cells by 15% at a frequency of 0.5 Hz for 1, 4, 12, 24, and 48 hours. Among the differentially expressed genes identified in the 1, 4, and 12 hour stretching groups, relatively few that changed at least 2-fold in expression were shared by all three groups (Supplementary Table 2).

**Figure 1:**
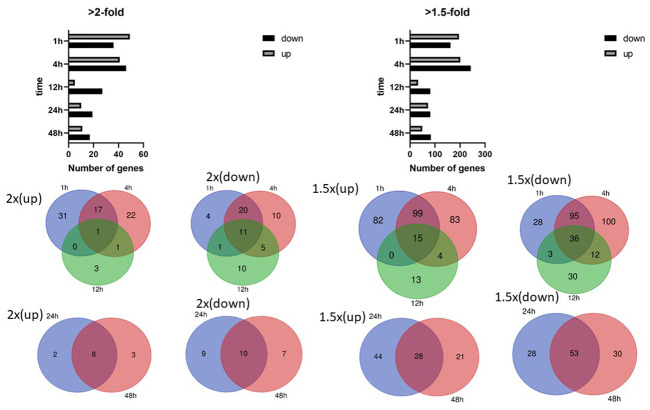
**Differentially expressed genes in human cardiomyocytes.** Numbers of differentially expressed genes after cyclic stretching: (**A**) Genes showing an at least 2-fold difference in expression; (**B**) genes showing an at least 1.5-fold difference in expression. Venn diagrams show the overlapping genes with at least 2-fold and 1.5-fold differences in expression.

In contrast, a larger number of genes showing a 2-fold or greater change in expression were shared by both the 24 and 48 hour stretching groups (Figure 1). Expression of the Serpin Family E Member 1 (SERPINE1) gene, which encodes plasminogen activator inhibitor 1 (PAI-1) and is involved in blood clotting, was significantly increased at all the time points. SERPINE1 activity is especially crucial in injuries in which inhibition of fibrinolysis is necessary to protect the body from excessive blood loss.

### Short- and long-term cyclic stretching differentially alter gene expression profiles

**Figure 2:**
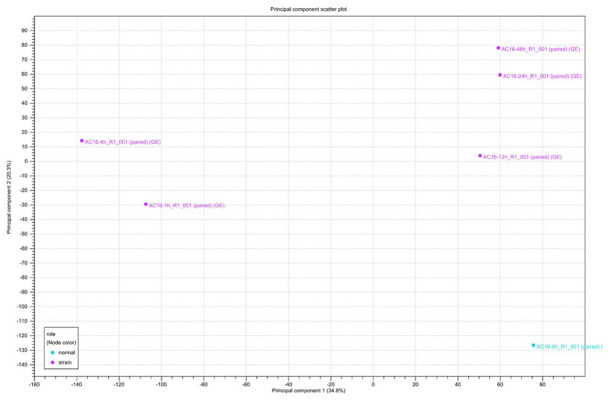
**Principal component analysis (PCA) of RNA-seq data.** Gene expression changes were investigated after 0, 1, 4, 12, 24, and 48 hours of cyclic stretching. PCA was performed using normalized RNA-Seq data for genes differentially expressed in one pairwise comparison: 0 h vs. other time points.

Clustering and principal component analysis are useful techniques for analyzing gene expression data that involve many complex biological networks. Principal component analysis revealed that cells could be separated into groups based on the duration of cyclic mechanical stretching treatment. As shown in Figure 2, principal component analysis revealed that human cardiomyocytes could be separated into long-term (12, 24, and 48 hours) and short-term (1 and 4 hours) stretch effect groups. Gene expression profiles of cells treated with cyclic stretching for 1 and 4 hours had similar metrics which differed from those observed in the static condition (zero hour) group, which was not subjected to any cyclic mechanical stretching treatment (Figure 2).

### Functional and pathway analysis of differentially expressed genes

**Figure 3:**
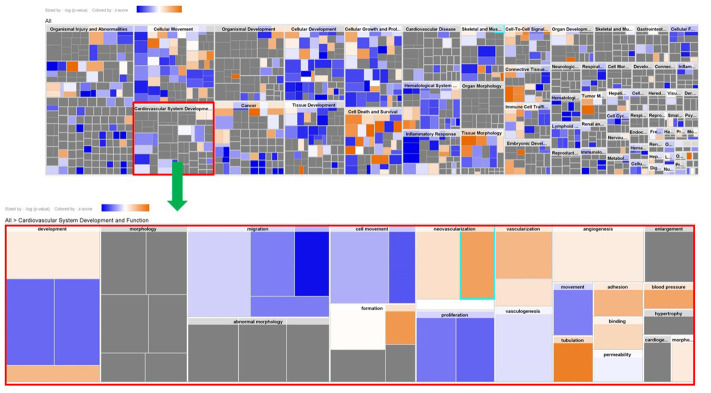
**Expression analysis heatmap for disease and functional pathways.**

**Figure 4:**
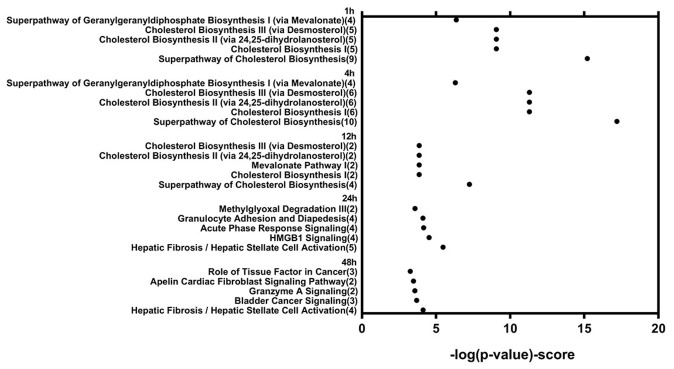
**Functional analysis of differentially expressed genes in human cardiomyocytes after 1, 4, 12, 24, and 48 hours of cyclic stretching.**

**Figure 5:**
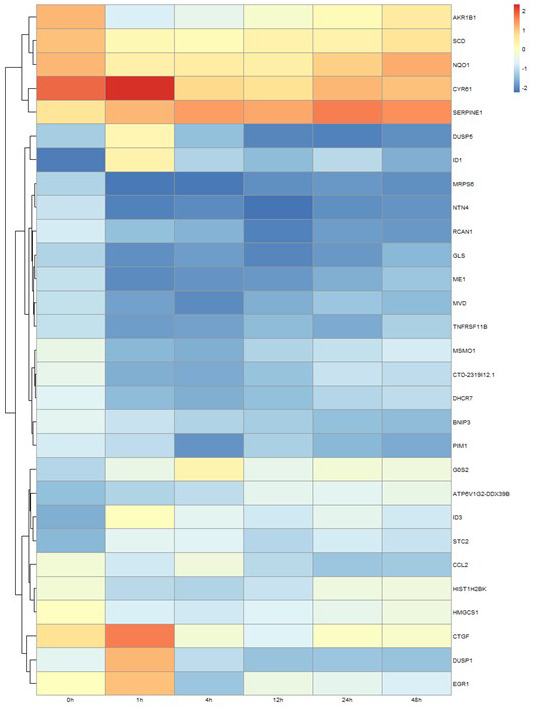
**Differential expression profile of 29 genes after 1, 4, 12, 24, and 48 hours of cyclical stretching in human cardiomyocytes.** The heat map diagram shows expression changes for 29 genes that were altered in cardiac myocytes in response to at least three time points of mechanical stretching.

Genes showing cyclic stretching-dependent differential expression (at least 2-fold difference) were further examined in a functional enrichment analysis using Ingenuity Pathway Analysis for diseases and biological functions. As shown in Figure 3, cyclic stretching-associated genes were enriched in organismal injury and abnormalities (*p* = 1.92E-29), cardiovascular system development and function (*p* = 1.36E-20), and cellular movement (*p* = 6.61E-20). Cardiovascular system development and function included the development of vasculature, migration, neovascularization, and vascularization (Figure 3). Among the biological pathways, lipid metabolism was enriched in the 1-, 4-, and 12-hour cyclic stretching treatment groups. Genes that were differentially expressed response to 24 and 48 hours of cyclic stretching were enriched in tissue fibrosis and the inflammation pathway. The top five most significantly altered biological pathways at each time point are shown in Figure 4. Twenty-nine cyclic stretching-associated genes that were significantly differentially expressed in at least three of the time point groups are shown in the heatmap in Figure 5.

### Validation of cyclic stretching-associated gene expression by real-time PCR

**Figure 6:**
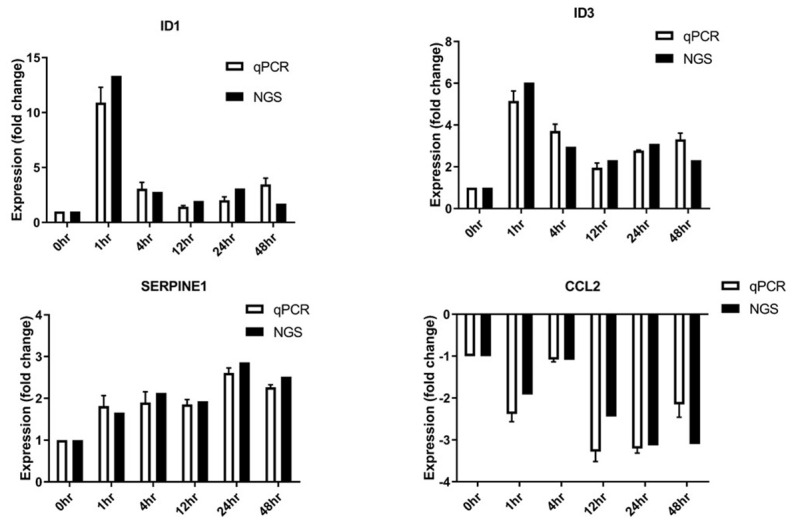
**Differentially expressed genes validated by RT-qPCR.** SERPINE1, ID1, ID3, and CCL2 mRNA levels were analyzed (n = 3 in all groups).

Short-term stretching for 1, 4, and 12 h significantly altered expression of genes associated with cholesterol biosynthesis, while long-term stretching for 24 and 48 h primarily affected expression of genes related to cell migration and enzymatic activity (Figure 4); other genes for which expression was altered by cyclic stretching are shown in Figure 5. Differentially expressed genes for which treatment effects differed between the short-term and long-term stretching groups are shown in Supplementary Table 3. Among them, ID1 (short-term stretching effect), ID3 (short-term stretching effect), SERPINE1 (long-term stretching effect), and CCL2 (long-term stretching effect) were selected for qPCR validation. The amount of strain used was relevant to cardiac arrhythmia; acute arrhythmia affects cholesterol biosynthesis and metabolism, while prolonged arrhythmia decreases cell viability and activates various cellular signaling pathways. We found that ID1 expression increased significantly increased after 1 h of stretching, while ID3 expression increased significantly after 1, 4, 12, 24, and 48 h of stretching (Figure 6). CCL2, which is involved in injury-associated inflammatory processes in cardiac cells, decreased after all durations of cyclic stretching treatment (Figure 6).

## DISCUSSION

Bone morphogenetic protein (BMP) signaling is involved in cyclic stretch-induced aortic valve calcification. Overproduction of BMP4 is pro-inflammatory in vascular cells and has been linked to hypertension [12, 13] and increases ID1 expression. ID1 expression is regulated by methylated cholesterol myristate, which has been linked to mesenchymal stem cells (MSCs) and neuronal cell survival [14, 15]. In addition, cyclic stretching is involved in the induction of lipotoxicity during atrial myocyte enlargement [16]. Together, these findings suggest that cyclic stretching greatly impacts the BMP4 signaling-dependent cholesterol biosynthesis pathway. Although it is acknowledged that disturbances in cholesterol biosynthesis may lead to cardiac disease [17], the effects of mechanical stretch on cholesterol biosynthesis remain unclear.

The ID1 gene, which encodes DNA-binding protein inhibitor ID-1, is an early downstream target of BMP4 signaling in various cells, including endothelial cells and embryonic stem cells [15, 18]. For example, ID1 is activated by Activin A receptor-like Type 1 (ALK1), which is a target gene of BMP [12]. Furthermore, ID1 activity during BMP receptor II (BMPRII) transition in lipid rafts may lead to abnormal cell growth [19]. However, whether ID gene expression is affected by mechanical stretch remains unclear. In this study, we found that cyclic stretching was associated with changes in the cholesterol biosynthesis signaling pathway. Specifically, ID1 gene expression increased after one hour of cyclic stretching. Furthermore, increases in expression of the SERPINE1 and ID3 genes, which are associated with obesity, and a decrease in pro-inflammatory CCL2 gene expression highlighted the impact of mechanical stretch on the cholesterol biosynthesis pathway.

Because ID1 plays roles in cell growth, differentiation, and apoptosis [20, 21], mechanical stretch might also affect these processes by altering its expression. Increases in CYR61 gene expression, which affects cell adhesion and proliferation, after one hour of stretching indicate that it may impact those processes as well. Such increases in anti-apoptotic and pro-survival genes at early time points may help maintain various cellular functions and signaling mechanisms. In contrast, after 24 and 48 hours of mechanical stretching, gene expression increases shifted from the cholesterol biosynthesis pathway to disease pathways, including acute phase response, cancer signaling, and hepatic fibrosis. Furthermore, no significant differences in genes showing differential expression were observed between the 24- and 48-hour mechanical stretching groups, indicating that 24 hours of stretching induced a relatively stable pro-inflammatory response that followed changes to the cholesterol-signaling pathway.

In this study, we examined transcriptional profile changes in human cardiomyocytes in response to cyclic stretching for the first time. Expression of a gene that has been previously linked to BMP signaling was altered after one hour of short-term cyclic stretching. BMPs exert both paracrine and autocrine effects and can also bind to type 1 or type 2 receptors to trigger intracellular signaling [22]. Endothelial cells, fibroblasts, and vascular smooth muscle cells express BMP2 and BMP4, which are important for heart development in vertebrates [23, 24]. Increases in BMP4 expression are associated with increased cholesterol levels, and BMP4 targets the ID1 promoter to drive anti-apoptotic cellular activities [25, 26]. Cholesterol myristate mediates ID1 activity, which promotes activation of B-cell lymphoma-extra large (Bcl-xL), leading to inhibition of cytochrome c release from mitochondria and decreased caspase activity [15]. In order to effectively promote survival, this BMP4-ID1 signaling cascade must be activated before the pro-inflammatory response is activated. The balance between cholesterol-mediated survival and pro-inflammatory responses can therefore determine whether normal or disease states are maintained.

While we examined the effects of mechanical load in cardiomyocytes, it also affects other cell types, including vascular, neuronal and bone cells, *in* *vivo* [27–30]. Our results highlight the crucial effects mechanical stretching can have on the cholesterol biosynthesis pathway as well as in cardiac disease. Furthermore, prolonged stretching could be a key cause of cardiomyopathy and various cardiovascular diseases. A better understanding of cholesterol metabolism could provide additional insights into the effects of mechanical stretching and the prevention of cardiac diseases in general.

Some limitations should be considered when interpreting the results of this study. Although the human cardiomyocyte cell line we used to examine the effects of cyclic stretching reflects human cardiac physiology relatively well, the cells will eventually undergo spontaneous differentiation and lose their morphology after long-term culture. In addition, they do not undergo spontaneous contraction or relaxation. However, since they can be serially passaged *in* *vitro* and further differentiated when cultured under mitogen-free conditions, human cardiomyocytes are ideal for developmental and pathological studies [31, 32]. Nevertheless, the results in this study pertain to proliferating cardiomyocytes, and additional studies will be necessary to determine their relevance to other types of cardiac cells.

In conclusion, in this study we describe expression profiles for genes that are differentially expressed in response to cyclic stretching in cardiomyocytes. Furthermore, we confirmed alterations in ID1 and SERPINE mRNA levels, which are likely involved in the cholesterol biosynthesis pathway. These results may improve our understanding of the cellular signaling pathways underlying cardiac injury.

## MATERIALS AND METHODS

### Cyclic stretching

Human ventricular cardiomyocyte cells (AC16) were maintained in DMEM/F12 (GeneDireX Incorporation, Taiwan) supplemented with 10% fetal bovine serum (Fisher Scientific, Pittsburgh, PA, USA), 100 IU/mL penicillin (Sigma-Aldrich) and 100 μg/mL streptomycin (Sigma-Aldrich). Cells were incubated in a humidified atmosphere at 37°C with 5% CO_2_. The cells were passaged every 3 to 4 days, and passages 2 to 10 were used in this study.

### Stretching device

The cells were stretched using a stretching device from ARTEMIS ATMS Boxer (TAIHOYA Corporation, Kaohsiung, Taiwan). Cells were seeded on polydimethylsiloxane (PDMS) pre-coated with collagen type 1 overnight. The next day, cells were stretched at 15% strain and a frequency of 0.5 Hz for at 0, 1, 4, 12, 24, or 48 h.

### RNA isolation

Total RNA was isolated using Trizol (Invitrogen) according to the manufacturer’s instructions. Briefly, chloroform was added to the mixture, followed by thorough mixing and centrifugation at 12,000 G for 15 min at 4°C. The clear supernatant was collected in a new microtube and isopropanol was added, followed by thorough mixing and centrifugation at 12,000 G for 15 min at 4°C. Finally, the RNA was washed with 75% ethanol and centrifuged at 7000 G for 5 min at 4°C. The RNA pellet was air-dried, reconstituted in DEPC water, and stored at -80°C.

### RNA sequencing

RNA sample quality was assessed using a NanoDrop spectrophotometer (Thermo Fisher Scientific Inc.) and a Bioanalyzer 2100 (Agilent Technologies, Santa Clara, CA, USA). The total RNA was subjected to NGS library construction using the MGIEasy RNA Library Prep Set (MGI Tech Co., Ltd., China). The quality and the average length of the sequence library for each sample were assessed using either the Bioanalyzer (Agilent Technologies, Santa Clara, CA, USA) or the DNA 1000 kit. The indexed samples were pooled equimolarly and sequenced on the BGISEQ-500 platform (50 bases, single-end reads) (BGI, China). The RNAseq data are publicly available on NCBI's Sequence Read Archive (SRA) database. (Bio-project: PRJNA612764).

### Sequencing data analysis

Clean reads were generated from raw sequencing reads, which were filtered to remove the adapters, unknown biases, and low quality reads. Clean reads were aligned to the reference genome build using Bowtie2 v2.2.5 [33]. For gene expression analysis, the matched reads were calculated and then normalized to FPKM using RSEM [34]. PCA analysis was performed with all samples using CLC Genomics Workbench (QIAGEN company, Redwood City, CA, USA), and diagrams were drawn with ggplot2 with functions of R. The gene expression data are listed in Supplementary Table 1.

### Functional enrichment analysis

Pathway enrichment was analyzed using Ingenuity Pathway Analysis (IPA) (QIAGEN company, Redwood City, CA, USA). The core analysis was based on a 2-fold change minimum in the gene profile. Significant pathways (*p* < .05) were identified using the database.

### Real-time PCR (RT-qPCR)

The differentially expressed genes identified by RNAseq were confirmed by performing quantitative real-time polymerase chain reaction (RT-qPCR) for four selected genes – ID1, ID3, CCL2, and SERPINE1. First, first-strand cDNA was generated from 0.1 μg of RNA using a ProtoScript® II First Strand cDNA Synthesis Kit (New England Biolabs, Inc., USA) in the presence of oligo-dT primers. After cDNA amplification, qPCR reactions were run on an ABI™ StepOne™ Real-Time PCR System (Applied Biosystems, Foster City, CA, USA) with KAPA SYBR® FAST Master Mix (2X) ABI Prism™ (KAPA BIOSYSTEMS, Boston, Massachusetts, United States). Gene expression levels were normalized to the expression of the internal housekeeping gene GAPDH. Relative quantification was calculated using the 2−ΔΔCT method. The sequences of the primers used are listed in Supplementary Table 1.

## Supplementary Material

Supplementary Tables 1, 3

Supplementary Table 2
