# Supplementary material for: Systems biology approach to exploring the effect of cyclic stretching on cardiac cell physiology
Source: Aging (Albany NY). 2020 Aug 5;12(16):16035–45. doi: 10.18632/aging.103465 (PMC7485730; doi:10.18632/aging.103465)
Supplement: Supplementary Tables 1, 3 [file aging-12-103465-s002..pdf]

SUPPLEMENTARY TABLES

Supplementary Table 1. List of primer sequences used for real time PCR analysis in this study.

Gene name	sequence (5'- 3')
ID1-F	GTAAACGTGCTGCTCTACGACATGA
ID1-R	AGCTCCAACTGAAGGTCCCTGA
ID3-F	TCATCTCCAACGACAAAAGG
ID3-R	ACCAGGTTTAGTCTCCAGGAA
GAPDH-F	TGCACCACCAACTGCTTAGC
GAPDH-R	GGCATGGACTGTGGTCATGAG
CCL2-F	GCTCATAGCAGCCACCTTCATTC
CCL2-R	GGACACTTGCTGCTGGTGATTC
SERPINE1-F	CACAAATCAGACGGCAGCACT
SERPINE1-R	CATCGGGCGTGGTGAACTC

F: Forward primer. R: reverse primer.

Please browse Full Text version to see the data of Supplementary Tables 2

Supplementary Table 2. The gene expression profile with a 2-fold significance at each time point.

Supplementary Table 3. The consistency DEG gene list in the short-term and long term stretch effect in the human cardiomyocytes.

Short term	Long term
AKR1B1	AKR1B1
COL6A3	ATP6V1G2-DDX39B
CTD-2319I12.1	BNIP3
DDX21	CCL2
DHCR7	CTGF
ERRFI1	CYR61
F3	DUSP1
FOSL1	DUSP5
HIST1H2BK	G0S2
HMGCS1	HMOX1
ID1	ID3
ID3	MMP14
IL11	MMP2
MAFF	NTN4
MAFK	PIM1
ME1	RCAN1
MOK	SEMA7A
MRPS6	SERPINE1
MSMO1	
MVD	
NQO1	
ODC1	
RBM3	
RRS1	
SCD	
SDC4	
SERPINB2	
STC2	
TAGLN	
TM4SF1	
