# Supplementary material for: Systems biology approach to exploring the effect of cyclic stretching on cardiac cell physiology
Source: Aging (Albany NY). 2020 Aug 5;12(16):16035–45. doi: 10.18632/aging.103465 (PMC7485730; doi:10.18632/aging.103465)
Supplement: Supplementary Table 2 [file aging-12-103465-s001..pdf]

Supplementary Table 2. The gene expression profile with a 2-fold significance at each time point.

a. 0 vs 1 h

Feature ID	Experiment - Fold Change (original values)	0 hr - AC16-0h_R1_001 (paired) (GE)_selection - Expression values	1 hr - AC16-1h_R1_001 (paired) (GE)_selection - Expression values
ACAT2	-2.17463	81.69699	37.56821
ADAMTS1	2.606454	31.07067	80.98428
AKR1B1	-7.74557	350.2118	45.21446
AMOTL2	3.121124	23.6311	73.75562
ANGPTL4	-2.02447	41.47979	20.48921
ASB1	2.270434	11.26891	25.58531
BHLHE40	3.467985	19.28587	66.88311
C14orf1	-2.49732	30.19344	12.09031
CHMP1B	2.205131	21.02623	46.36559
COL1A2	-2.69033	261.2981	97.12481
COL3A1	-2.18036	262.1411	120.2282
CTD-2319I12.1	-3.17745	58.24561	18.33091
CTGF	2.845878	207.2336	589.7616
CXCL12	-2.11967	69.42758	32.754
CYP51A1	-2.00404	36.85394	18.38982
DDX21	2.106769	35.45006	74.6851
DHCR7	-2.38849	50.64358	21.20322
DRAM1	-2.16739	24.91347	11.49468
DUSP1	6.541208	53.17508	347.8293
DUSP5	4.852638	27.20033	131.9933
EGR1	2.982806	105.7189	315.339
ENC1	2.097826	12.52134	26.2676
ERRFI1	3.904868	11.72734	45.7937
ETS2	2.09441	22.87182	47.90298
F3	3.447532	34.50163	118.9455
FAM114A1	-2.00471	38.9013	19.40491
FDFT1	-2.41426	100.3698	41.57373
FDPS	-2.24963	143.8791	63.95685

FHL1	-2.06648	38.18014	18.47591
FOSL1	4.877606	36.21552	176.6451
GADD45A	2.235088	25.21211	56.35129
GLS	-2.26866	29.63961	13.06481
GPRC5A	3.622686	13.27621	48.09553
HIST1H2BK	-2.38153	77.27431	32.44733
HMGCS1	-2.30938	103.5093	44.82118
ID1	13.33936	10.56022	140.8665
ID3	6.036495	17.82203	107.5826
IDH1	-2.0105	33.32784	16.57691
IL11	4.769934	22.19423	105.865
ITPRIP	3.072336	10.59403	32.5484
JUNB	2.199929	50.21775	110.4755
KDELR3	-2.13895	81.545	38.12379
KLF10	3.1374	14.46563	45.38448
KLF6	2.627525	28.19201	74.07522
LUM	-4.02898	42.10405	10.4503
LY6K	2.362486	11.85449	28.00606
MAFF	4.443254	13.81408	61.37948
MAFK	3.086123	10.4585	32.2762
MAGED2	-2.13943	91.80914	42.91296
MCL1	2.087714	70.75054	147.7069
ME1	-2.90736	35.70866	12.28218
MOK	2.240017	13.62858	30.52825
MRPS6	-2.78865	29.05564	10.41925
MSMO1	-2.98619	61.13105	20.47126
MT1X	2.365246	21.668	51.25015
MVD	-2.32526	35.87646	15.42898
MYC	3.757436	19.83376	74.52406
NQO1	-2.26804	345.219	152.2106
NTN4	-3.30503	37.26309	11.27467
ODC1	2.976129	21.33575	63.49794
PCSK9	-2.69049	65.52329	24.35368
PHLDA1	2.375768	13.43059	31.90796
PINK1	-2.18075	28.92306	13.26292
PMAIP1	2.069217	16.89855	34.96676
PPP1R15A	3.328939	24.48713	81.51616
QPRT	-2.32566	46.76273	20.10727
RBM3	2.189415	31.203	68.31631

RGMB	2.189505	13.84475	30.31315
RHOB	3.517249	28.43928	100.028
RP11-396K3.1	-2.24129	25.99418	11.59785
RRS1	2.009035	15.94702	32.03812
SCD	-2.4573	304.8016	124.0391
SDC4	2.03778	60.38483	123.051
SERPINB2	4.360033	10.9186	47.60547
SGK1	3.761052	12.38366	46.57558
SQLE	-2.13982	49.2196	23.00171
STC2	2.6455	20.21855	53.48818
TAGLN	2.15126	23.24849	50.01355
TIPARP	2.75525	12.35222	34.03345
TM4SF1	2.335403	18.43261	43.04758
TNFRSF11B	-2.40805	35.58808	14.77877
TSC22D1	2.287374	11.72614	26.82206
TUBA1A	-2.59573	154.7863	59.631
YRDC	2.078385	10.67398	22.18463
ZFP36	2.368129	15.51095	36.73193

b. 0 vs 4 h

Feature ID	Experiment - Fold Change (original values)	0 hr - AC16-0h_R1_001 (paired) (GE)_selection - Expression values	4 hr - AC16-4h_R1_001 (paired) (GE)_selection - Expression values
ACAT2	-2.31653	81.69699	35.26695
ADM	-2.81093	64.46429	22.93348
AKAP12	2.753147	16.44593	45.27807
AKR1B1	-5.91543	350.2118	59.20314
ATP13A3	2.200955	15.65591	34.45796
C14orf1	-2.35691	30.19344	12.81061
CARD10	2.215217	11.08944	24.56551
CBFB	2.228991	13.14279	29.29517
CCDC85B	-2.00033	39.45163	19.72253
CLUH	2.122967	17.4407	37.02603
COL1A2	-2.695	261.2981	96.95656
COL3A1	-2.24571	262.1411	116.7295
COL6A3	2.239325	40.70704	91.1563
CTD-2319I12.1	-3.3264	58.24561	17.5101
CTGF	-2.72298	207.2336	76.10559
CYP51A1	-2.09336	36.85394	17.60513
CYR61	-2.96814	721.8393	243.1958
DDX21	2.689998	35.45006	95.36059
DHCR7	-2.80912	50.64358	18.02825
DKK1	2.331932	59.02423	137.6405
EBP	-2.05175	56.90401	27.73434
EGR1	-4.40167	105.7189	24.0179
ERRFI1	2.316143	11.72734	27.16219
F3	2.505848	34.50163	86.45584
FADS2	-2.18905	69.00272	31.52172
FAM114A1	-2.08163	38.9013	18.68789
FAM57A	2.039366	12.63105	25.75933
FDFT1	-2.52554	100.3698	39.7419
FDPS	-2.41878	143.8791	59.48422
FHL1	-2.09757	38.18014	18.2021
FOSL1	2.679865	36.21552	97.05272

FZD2	-2.6558	44.71939	16.83841
G0S2	4.320591	31.23836	134.9682
HCFC1R1	-2.20024	38.32071	17.4166
HIST1H2BK	-2.55786	77.27431	30.21055
HMGCS1	-2.55748	103.5093	40.47313
ICAM1	2.138561	12.417	26.55452
ID1	2.782661	10.56022	29.3855
ID3	2.96481	17.82203	52.83892
IDH1	-2.26392	33.32784	14.72132
IER2	-2.85613	47.09764	16.49001
IL11	3.578611	22.19423	79.42451
KDELR3	-2.05012	81.545	39.77567
LUM	-3.46007	42.10405	12.16855
MAFF	2.662646	13.81408	36.78202
MAFK	2.239265	10.4585	23.41935
MAGED2	-2.15667	91.80914	42.56987
MAP2K3	2.222938	28.65764	63.70416
ME1	-2.58276	35.70866	13.8258
MOK	2.146085	13.62858	29.2481
MRPS6	-2.88643	29.05564	10.06628
MSMO1	-3.41294	61.13105	17.91154
MVD	-2.86243	35.87646	12.53355
NQO1	-2.04853	345.219	168.5204
NTN4	-3.02209	37.26309	12.33023
ODC1	3.034009	21.33575	64.73284
P4HA1	-2.09376	50.56541	24.1505
PCSK9	-2.93702	65.52329	22.30943
PIM1	-3.27535	43.94156	13.41584
PINK1	-2.09158	28.92306	13.82833
PLAUR	2.053784	17.04763	35.01216
QPRT	-2.86835	46.76273	16.30303
RBM3	2.34228	31.203	73.08618
RCAN1	-2.32346	44.06674	18.96598
RND3	2.068435	60.38562	124.9037
RRS1	2.004016	15.94702	31.9581
SCD	-2.61845	304.8016	116.4054
SDC4	2.89103	60.38483	174.5744
SERPINB2	10.15059	10.9186	110.8303
SERPINB8	2.281382	10.36114	23.63772

SERPINE1	2.133111	204.5433	436.3135
SLC20A1	2.208489	46.84671	103.4605
SLC3A2	2.020673	27.11501	54.79056
SLC7A5	2.850991	63.22504	180.254
SMAD3	2.614217	10.68501	27.93294
SNAI2	-2.1965	39.03722	17.77245
SQLE	-2.06995	49.2196	23.77812
STC2	2.52221	20.21855	50.99542
TAGLN	2.041956	23.24849	47.47239
TJP2	2.21243	12.49177	27.63716
TM4SF1	2.077793	18.43261	38.29916
TMSB4X	-2.04588	618.6757	302.4003
TNFRSF11B	-2.28409	35.58808	15.58088
TNFRSF12A	2.007761	83.32493	167.2965
TOMM20	2.149877	39.62653	85.19218
TUBA1A	-2.76992	154.7863	55.88118
WDR43	2.252282	11.60241	26.1319

c. 0 vs 12 h

Feature ID	Experiment - Fold Change (original values)	0 hr - AC16-0h_R1_001 (paired) (GE)_selection - Expression values	12 hr - AC16-12h_R1_001 (paired) (GE)_selection - Expression values
ADM	-2.06395	64.46429	31.2335
AKR1B1	-4.20729	350.2118	83.23919
AKR1C3	-3.98524	59.29942	14.87976
ATP6V1G2-DDX39B	2.556657	21.65055	55.35304
BNIP3	-2.01685	52.90728	26.23266
CCL2	-2.44187	77.59746	31.7779
COL6A3	2.062087	40.70704	83.94144
CTD-2319I12.1	-2.45589	58.24561	23.71666
CTGF	-4.32893	207.2336	47.87184
CYR61	-3.41196	721.8393	211.5617
DHCR7	-2.327	50.64358	21.76345
DUSP1	-2.24865	53.17508	23.6476
DUSP5	-2.34222	27.20033	11.61304
GADD45A	-2.15579	25.21211	11.69504
GLS	-2.47133	29.63961	11.9934
HIST1H1C	-2.19762	26.57137	12.09098
HIST1H2BK	-2.05092	77.27431	37.67792
HMGCS1	-2.16585	103.5093	47.79155
ID3	2.321129	17.82203	41.36723
IER3	-2.43856	444.4204	182.2467
IGFBP6	2.016992	17.67345	35.64719
INSIG1	-2.39042	130.285	54.50287
LAYN	-2.01858	23.01253	11.40035
ME1	-2.50514	35.70866	14.25414
MRPS6	-2.27229	29.05564	12.78696
MSMO1	-2.12004	61.13105	28.83489
MVD	-2.00496	35.87646	17.89389
NQO1	-2.21584	345.219	155.7961
RCAN1	-3.98948	44.06674	11.04572
SCD	-2.11594	304.8016	144.0502

SNAI2	-2.04113	39.03722	19.12533
TGFBI	2.0849	41.85123	87.25562

d. 0 vs 24 h

Feature ID	Experiment - Fold Change (original values)	0 hr - AC16-0h_R1_001 (paired) (GE)_selection - Expression values	24 hr - AC16-24h_R1_001 (paired) (GE)_selection - Expression values
AKR1B1	-3.00518	350.2118	116.536
AKR1C3	-2.76789	59.29942	21.42403
ATP6V1G2-DDX39B	2.393668	21.65055	51.82425
BNIP3	-2.4079	52.90728	21.97237
CCL2	-3.1312	77.59746	24.78201
CTGF	-2.10211	207.2336	98.58365
CYR61	-2.07074	721.8393	348.5902
DUSP1	-2.14352	53.17508	24.80735
DUSP5	-2.45314	27.20033	11.08795
FOS	-2.14762	31.23322	14.54319
G0S2	2.528052	31.23836	78.97218
GLS	-2.03065	29.63961	14.59609
HMOX1	2.157586	17.97391	38.78026
ID1	3.092643	10.56022	32.65897
ID3	3.105737	17.82203	55.35052
INSIG1	-2.09942	130.285	62.05764
MALAT1	-2.29657	48.55589	21.14282
MMP14	2.069486	43.54416	90.11405
MMP2	2.06404	52.88054	109.1476
NME2	-2.03038	34.50676	16.9952
NTN4	-2.81426	37.26309	13.24081
PIM1	-2.17168	43.94156	20.23392
RCAN1	-2.91566	44.06674	15.11381
SCD	-2.06483	304.8016	147.6161
SEMA7A	2.423314	18.97512	45.98268
SERPINE1	2.8626	204.5433	585.5256
STC2	2.13909	20.21855	43.2493
TNFRSF11B	-2.03233	35.58808	17.51102
UGCG	-2.01954	30.96527	15.33285

e. 0 vs 48 h

Feature ID	Experiment - Fold Change (original values)	0 hr - AC16-0h_R1_001 (paired) (GE)_selection - Expression values	48 hr - AC16-48h_R1_001 (paired) (GE)_selection - Expression values
AKR1B1	-2.00694	350.2118	174.5002
ATP6V1G2-DDX39B	2.792779	21.65055	60.46521
BNIP3	-2.54107	52.90728	20.82089
CCL2	-3.10006	77.59746	25.03099
CTGF	-2.36536	207.2336	87.61185
CYR61	-2.36522	721.8393	305.1887
DUSP1	-2.31083	53.17508	23.01125
DUSP5	-2.10908	27.20033	12.89678
EGR1	-2.319	105.7189	45.58822
G0S2	2.251148	31.23836	70.32217
H1F0	-2.05104	172.843	84.27103
H1FX	-2.28415	123.1371	53.90938
HMOX1	3.502695	17.97391	62.95713
ID3	2.316912	17.82203	41.29207
IER3	-2.21388	444.4204	200.7427
JUND	-2.51194	33.539	13.35183
MFAP4	2.267021	11.53735	26.15541
MMP14	2.184996	43.54416	95.14383
MMP2	2.63412	52.88054	139.2937
MRPS6	-2.19701	29.05564	13.22509
NTN4	-2.72563	37.26309	13.67137
PIM1	-2.52337	43.94156	17.41385
RCAN1	-3.03048	44.06674	14.5412
S100A10	-2.10959	134.8078	63.9025
SEMA7A	2.184576	18.97512	41.45259
SERPINE1	2.516912	204.5433	514.8174
SQSTM1	2.473543	38.11028	94.2674
THBS1	2.332123	71.16483	165.9651
